# Feasibility of Magnetic Resonance Imaging–Guided Pulmonary Artery Stenting in a Commercial Wide-Bore 0.55-T Scanner

**DOI:** 10.1016/j.jacbts.2026.101562

**Published:** 2026-05-18

**Authors:** Yixuan Liu, John M. Kelly, Jason Swinning, Yingmin Liu, Matthew Joseph, Ning Jin, Jianing Pang, Florian Maier, Axel J. Krafft, Nathan A. Ooms, Jesse Roll, Joshua Krieger, David C. Gross, Orville Bramwell, Lucien de Mos, Paul Borm, Orlando P. Simonetti, Aimee K. Armstrong

**Affiliations:** a Department of Biomedical Engineering, The Ohio State University, Columbus, Ohio, USA; b Heart Center, Nationwide Children’s Hospital, Columbus, Ohio, USA; c Department of Pediatrics, The Ohio State University, Columbus, Ohio, USA; d Division of Cardiovascular Medicine, Department of Internal Medicine, The Ohio State University, Columbus, Ohio, USA; e Center for Regenerative Medicine, Abigail Wexner Research Institute at Nationwide Children’s Hospital, Columbus, Ohio, USA; f Dorothy M. Davis Heart and Lung Research Institute, The Ohio State University, Columbus, Ohio, USA; g Siemens Medical Solutions USA Inc., Malvern, Pennsylvania, USA; h Siemens Healthineers AG, Erlangen, Germany; i Cook Advanced Technologies, West Lafayette, Indiana, USA; j College of Health and Human Science, Purdue University MRI Facility, West Lafayette, Indiana, USA; k Cook Medical, Bloomington, Indiana, USA; l MED Institute, West Lafayette, Indiana, USA; m Nano4Imaging GmbH, Düsseldorf, Germany; n Department of Radiology, The Ohio State University, Columbus, Ohio, USA

**Keywords:** congenital heart disease, MRI-guided interventions, pulmonary artery stenting

## Abstract

Real-time pulmonary artery (PA) stenting under magnetic resonance imaging (MRI) guidance may eliminate radiation exposure during complex vascular interventions. This study evaluated the feasibility of performing PA stenting in a swine model using a commercially available 0.55-T MRI system with ferumoxytol-enhanced imaging. Ten juvenile pigs underwent attempts at MRI-guided PA stent deployment using stainless-steel balloon-expandable stents. Stenting was successful in 7 animals, with all successes achieved after switching from femoral to external jugular venous access. Real-time gradient echo imaging provided sufficient visualization for catheter navigation, device delivery, and stent deployment. Postdeployment phase contrast imaging demonstrated flow continuity, minimal artifact, and trivial pressure gradients across the stent (0.2 ± 0.12 mm Hg). Peak velocities increased by 21.2 ± 7.0 cm/s distal to the stent, confirming preserved patency. These findings demonstrate the technical feasibility of CMR-guided PA stenting at 0.55-T and support the potential for future radiation-free PA interventions.

## Highlights

• PA rehabilitation is a common and radiation-intensive intervention in patients with CHD. CMR-guided intervention using mid-field (0.55-T) MRI potentially offers a radiation-free alternative. Lower magnetic field strength reduces the risk of device heating, enabling the use of standard catheterization equipment with minor modifications.
• This study demonstrates the feasibility of real-time CMR-guided branch PA stenting in a large animal model using a commercial 0.55-T scanner, supporting the viability of radiation-free PA interventions and expanding the scope of mid-field iCMR.
• The study outlines the need for further refinement of interventional devices and imaging techniques to enhance the clinical utility of mid-field iCMR, including the development of marker identification and advanced rapid acquisition techniques.

## Visual Abstract

**Figure:**
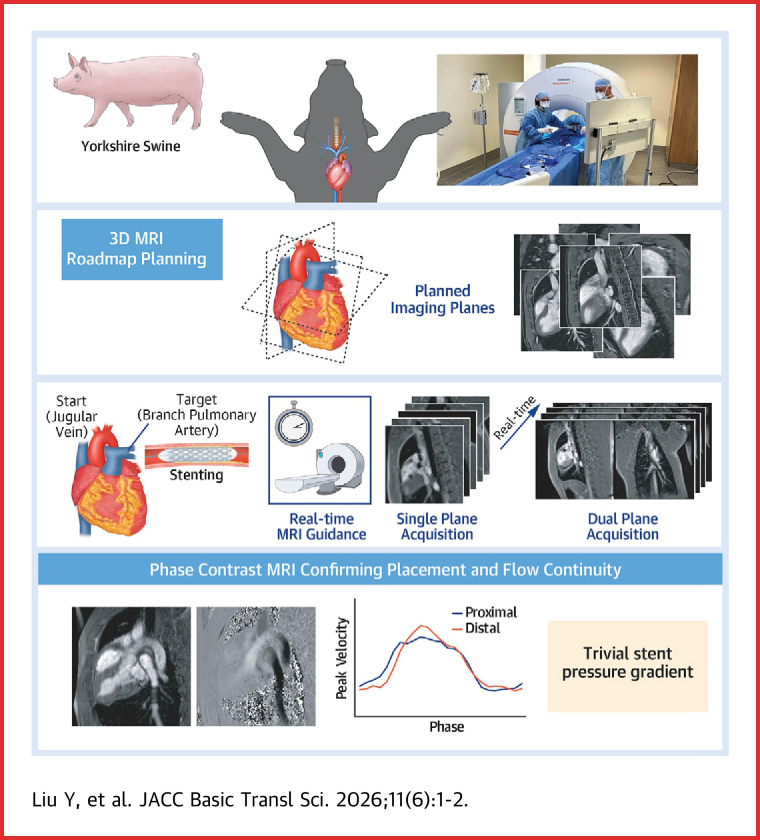

Interventional cardiovascular magnetic resonance (iCMR) has emerged over the past two decades as a promising approach for guiding cardiovascular catheterization and interventions without ionizing radiation.^1^ The first entirely magnetic resonance imaging (MRI)–guided cardiac catheterization in a patient with congenital heart disease (CHD) was reported nearly 20 years ago by Dr Reza Razavi and colleagues at Guy’s Hospital in the United Kingdom,^2^ and since then a growing number of centers have explored iCMR for diagnostic catheterization and select interventions.

The feasibility of performing various catheter-based interventions under real-time MRI guidance has been previously demonstrated in animal models, including balloon angioplasty of branch pulmonary arteries (PAs) and stenting of the main PA.^1^^,^^3^, ^4^, ^5^ Over 20 years ago, Kuehne et al^4^ reported magnetic resonance imaging (MRI)–guided deployment of self-expanding endovascular stents in the main PA and pulmonary valve of swine on a 1.5-T MRI scanner, highlighting that even complex outflow tract interventions could be performed under MRI guidance.^4^ Nearly all prior iCMR studies have been performed at 1.5-T and 3-T to leverage the superior signal-to-noise ratio (SNR), high-performance gradient systems, and fast imaging capabilities of these systems.^1^^,^^4^^,^^6^ However, translation of iCMR beyond preclinical studies and controlled research settings has been limited, primarily due to the safety challenge of device heating associated with high-field (1.5-T and above) imaging and the lack of safe interventional devices that are also visible by MRI.^1^ These factors have prevented widespread adoption of iCMR, which has further limited industry interest in producing iCMR-compatible devices.

Some early investigations explored the potential for iCMR on “open” scanners with field strengths as low as 0.2-T.^7^ While those systems offered greater patient accessibility and reduced risk of device heating, the overall imaging performance of these early low-field systems was poor by modern standards, and iCMR at low field strengths was never widely adopted. More recently, mid-field MRI at 0.55-T has gained attention as a novel platform for interventional procedures that may overcome some high-field limitations.^1^^,^^8^ At this field strength, radiofrequency-induced heating of intravascular devices is substantially reduced, enabling the safe utilization of some standard, commercially available guidewires and catheters designed for use with x-ray fluoroscopy.^9^ This potential to use standard catheterization devices, either as is or with minor modifications, could greatly increase the practical applicability of iCMR. We recently demonstrated the technical feasibility of iCMR on a commercially available 0.55-T scanner with 80 cm diameter patient bore using standard catheterization devices, some of which had undergone minor modifications to improve visibility.^8^ Successful real-time MRI-guided right and left heart catheterization and inferior vena cava (IVC) balloon angioplasty and stenting procedures were performed in this preclinical study, despite the lower field strength and reduced gradient performance of this system. Notably, the imaging was facilitated by ferumoxytol, an intravascular iron oxide contrast agent administered to enhance blood-pool signal by significantly shortening T1.^10^ This enabled clear real-time visualization of cardiovascular structures and devices using a spoiled gradient echo (GRE) sequence.^8^ While IVC stenting is a relatively simple procedure, these results confirmed that endovascular interventions can be successfully performed at mid field, taking advantage of reduced device heating and the improved access provided by the ultra-wide bore, even with the trade-off of lower intrinsic SNR and modest gradient performance compared with modern 1.5-T scanners. Our previous investigation of IVC stenting at 0.55-T set the stage to test more complex procedures, such as PA stenting.

Branch PA stenosis is common in patients with CHD, and balloon angioplasty and stenting of these lesions may account for up to 16% to 20% of all catheter-based interventions in this population.^11^^,^^12^ These PA rehabilitation procedures are among the highest radiation-producing cases performed in a congenital cardiac catheterization laboratory, and the patients undergoing these procedures typically require repeated diagnostic studies and procedures with ionizing radiation. CHD patients are at an increased risk of developing cancer,^13^ and this risk is higher in patients who have received high lifetime doses of medical radiation.^14^ Cardiac catheterization is one of the main contributors to cumulative effective dose in CHD patients and can contribute up to 1,800 times as much effective dose per examination than a standard radiograph.^14^ Thus, finding a way to eliminate radiation exposure during PA rehabilitation is critical to improving care for CHD patients. PA stenting in CHD is primarily performed using balloon-expandable stents, and real-time MRI-guidance of this procedure has never been reported in preclinical or clinical settings. We sought to test the feasibility of this procedure in a large animal model using a commercially available 0.55-T MRI scanner.

## Methods

### Equipment

All imaging was performed on a 0.55-T MRI system (MAGNETOM Free.Max; Siemens Healthineers AG) with an 80 cm diameter, 165 cm length patient bore, and maximum gradient amplitude and slew rate of 26 mT/m and 45 mT/m/ms, respectively. A 12-channel flexible surface coil placed anteriorly, combined with a posterior 6-channel surface coil, were used for image acquisition.

A third-party patient monitoring system (Expression MR400; Philips N.V.) was connected to the MRI machine to monitor the electrocardiogram, arterial oxygen saturation, and invasive blood pressure. This device was also used for cardiac synchronization of image acquisition, as the scanner lacks an integrated physiological triggering system. Hemodynamic recording equipment was not available. The interventional cardiologist viewed the images in real time on an MRI conditional 4K display monitor (Inroom Viewing Device; NordicNeuroLab) that was connected to the MRI console display via a fiber-optic cable. A Bluetooth audio headset with noise cancellation (B350-XT; BlueParrott) and limited ferromagnetic attraction was worn by the interventional cardiologist to provide communication with the team members in the control room. The headset was paired with a mobile phone placed in the waveguide opening between the control room and magnet room, and a desktop phone at the MRI console was used to call the paired phone. This system allowed seamless communication and did not generate any detectable radiofrequency interference.

### Animals

Juvenile Yorkshire pigs were used for the experiments under a protocol approved by the local Institutional Animal Care and Use Committee (reference number 2021A00000076, approval date September 13, 2021). Prior to arrival at the MRI facility, the pigs were placed under general anesthesia, intubated, and maintained on 2% to 3% isoflurane and 50% inspired O_2_ during the studies. Following anesthesia induction, 16-F sheaths were inserted via cutdown into the right femoral vein in the first 2 animals and into the right external jugular vein in the remaining 8 animals. The first 6 pigs received 3 mg/kg ferumoxytol (Sandoz AG), and the remaining 4 pigs received a dose of 2 mg/kg. The contrast agent was diluted with saline at a ratio of 20:1 and infused at a slow rate of 200 mL/h prior to transporting the pigs to the MRI facility. When in the MRI scanner, each pig received amiodarone (100 mg intravenous) and magnesium sulfate (1 g intravenous) for arrhythmia prophylaxis and heparin (100 IU/kg intravenous) for thrombosis prophylaxis.

### Magnetic Resonance Imaging Protocol

A 3-dimensional (3D) volumetric interpolated breath-hold examination (VIBE) Dixon sequence was used to generate a 3D roadmap used for procedural planning. Two-dimensional GRE real-time sequences were employed for all MRI-guided interventions. The research interactive real-time imaging add-in (iMRI for MAGNETOM Free.Max, Research Software Package; Siemens Healthineers) enabled interactive control of slice position and orientation, allowing the operator to adjust the slice plane simultaneous with image acquisition and display. The add-in also supported toggling between single-plane and interleaved multiplane acquisitions, with the latter providing additional anatomical coverage at the cost of reduced temporal resolution.

All sequences were accelerated using GRAPPA (Generalized Autocalibrating Parallel Acquisition). For single-plane acquisitions, separate reference lines were acquired at the beginning of each scan and with each change in slice position. Single-plane scans were acquired at 4.3 frames per second (fps). In interleaved dual-plane acquisitions, integrated reference lines were used, which incurred a time penalty. The acquisition matrix was therefore reduced in dual-plane scans to help compensate for the reduced temporal resolution. Each plane required 191 ms to acquire, with the two planes updated alternately, resulting in an effective frame rate of 2.6 fps per slice. A phase-contrast GRE sequence was performed following stent deployment to assess stent patency. Detailed sequence parameters are provided in Table 1.

**Table 1:** Pulse Sequence Parameters Used

	Flip Angle (°)	TE (ms)	TR (ms)	RBW (Hz/pixel)	Temporal Resolution (ms)	Slice Thickness (mm)	Pixel Size (mm^2^)	Acquisition Matrix	Acceleration Rate
Single-plane IRT sequence	20	2.3	5.4	501	230	10	3.8 × 3.1	84 × 128	2
Dual-plane IRT sequence	20	2.3	5.3	501	191	10	4.4 × 3.5	78 × 96	3
3D VIBE Dixon	20	2.8/6.5	9.9	300	—	2	0.9 × 0.9	205 × 256	2
Phase contrast GRE	12	3.7	6.7	401	39	8	2.0 × 1.7	130 × 208	1

GRE = spoiled gradient echo; IRT = interactive real-time; RBW = receiver bandwidth; TE = echo time; TR = repetition time; VIBE = volumetric interpolated breath-hold examination.

### Scan workflow, devices, and procedures

**Figure 1:**
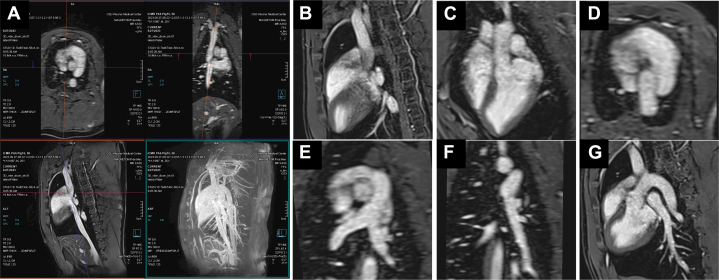
Roadmap Planning at the Beginning of the Procedure (A) The water image from the 3-dimensional volumetric interpolated breath-hold examination (VIBE) Dixon scan is loaded into the built-in multiplanar reformatting tool. Commonly used imaging planes for magnetic resonance–guided pulmonary artery stenting are reformatted from the 3-dimensional VIBE Dixon scan, including (B) superior vena cava (SVC), (C) 4-chamber, (D) right ventricular outflow tract, (E) pulmonary artery bifurcation, (F) left pulmonary artery coronal, and (G) left pulmonary artery sagittal views. Image sharpness varies due to the nonisotropic resolution of the VIBE acquisition (0.9 × 0.9 × 2.0 mm); oblique reformats intersecting the thicker slice dimension (C to F) exhibit partial volume blurring.

At the beginning of the procedure, 3D VIBE Dixon was acquired in the sagittal orientation. The acquired 3D images were imported into the multiplanar reformatting tool on the scanner console computer. Proximal left pulmonary artery (LPA) and right pulmonary artery (RPA) diameters were measured to select appropriate stent sizes. The imaging planes that would be required along the navigation track to maneuver the interventional devices to the branch PA were generated using the multiplanar reformatting tool and saved as roadmaps. These included the bicaval, 4-chamber, right ventricular outflow tract, PA bifurcation, LPA, and RPA views (Figure 1). These images were loaded into the research interventional MRI User Interface Extension (iMRI UI Extension, Research Software Package; Siemens Healthineers) on the scanner console to enable real-time interactive navigation between slice planes during image acquisition.

A 7-F Arrow wedge catheter (Teleflex) with a CO_2_-filled balloon and a 0.035-inch EmeryGlide guidewire (Nano4Imaging) inside of the catheter lumen were directed from the vena cava into either the RPA or LPA under real-time guidance, using a single-plane interactive real-time sequence. The 3 passive MagnaFy MR-visible markers near the tip of the EmeryGlide guidewire (Nano4Imaging) and the CO_2_-filled balloon facilitated visual tracking of the wire and catheter, as the wire was manually pinned inside the catheter. The imaging sequence was then switched to an interleaved 2-plane acquisition displaying both the bicaval and branch PA views. With the catheter in the distal lower lobe branch PA, the EmeryGlide guidewire was exchanged for a prototype exchange length guidewire with 4 MR-visible passive markers (Cook Medical). The wedge catheter was removed over the MR-visible exchange length guidewire, and a prototype MR-visible Flexor sheath (Cook Medical) was advanced over the guidewire. Both the dilator and sheath tips were labeled with passive MR-visible markers, and the shaft of the sheath was also visible, particularly when perpendicular to main magnetic field (B0). When the sheath tip was determined to be in the distal branch PA, the dilator was removed over the wire.

Commercially available stainless steel (316L) 26 mm long Mega and Max LD stents (Medtronic) were manually crimped onto Z-Med balloons (NuMED Inc.) that had been labeled with 4 MagnaFy MR-visible markers: 2 at the shoulders (0.5 mm, 1 layer thick) and 2 at the balloon tips (0.5 mm, 3 layers thick). The balloon and stent combination were advanced over the wire within the sheath to the branch PA. The sheath was retracted to expose the stent and angioplasty balloon. Imaging was then switched back to a single-plane acquisition of the branch PA to improve temporal resolution. The stent was deployed by inflating the angioplasty balloon with 1% gadolinium to 8 atm. The angioplasty balloon was then deflated and removed along with the wire, completing the procedure. Procedural time was recorded from wedge catheter insertion to stent deployment to assess the technical feasibility of MRI guidance, excluding the logistical time required for general anesthesia induction and animal transportation. Table 2 summarizes the devices used for the PA stenting procedures and indicates whether each device was commercially available or customized.

**Table 2:** Devices Used

Commercially available
7-F Arrow wedge catheters (Teleflex)
MRI-conditional 0.035-inch EmeryGlide guidewire (Nano4Imaging)
Z-Med balloons (NuMED Inc.) with MagnaFy markers (Nano4Imaging)^a^
Max and Mega LD stainless steel stent (Medtronic)
Prototype
Prototype exchange length guidewire (Cook Medical)
Prototype MR-visible Flexor sheath (Cook Medical)

a Customized markers.

A post-procedural 3D VIBE Dixon scan was acquired to confirm stent placement. A 2-dimensional phase contrast GRE sequence was performed along the branch PA at the stent site, while through-plane velocity maps were acquired proximal and distal to the stent in 4 animals to evaluate velocity and flow through the stent. The through-plane phase contrast GRE images were analyzed using SuiteHeart (version 5.4; Neosoft). Peak velocity was measured directly in the PA branches proximal and distal to the stent. Pressure gradients at these locations were estimated using the modified Bernoulli equation.

## Results

Ten juvenile pigs weighing 74.6 ± 11.2 kg underwent the attempted PA stenting procedure. The procedure was unsuccessful in the first 3 animals. The first two procedures failed due to technical challenges of accessing the PAs from the femoral venous approach. In the first animal, the 60 cm prototype sheath was too short to reach the right ventricle. In the second animal, an atrial loop was needed to get the interventional wire to the branch PA, and the 90 cm sheath would not follow this wire course. The third animal developed bradycardia after sheath insertion and subsequently progressed to pulseless electrical activity and expired. After changing the venous access location to the external jugular vein for the subsequent animals, stent deployment was successful in the remaining 7 (3 in the RPA and 4 in the LPA).

**Figure 2:**
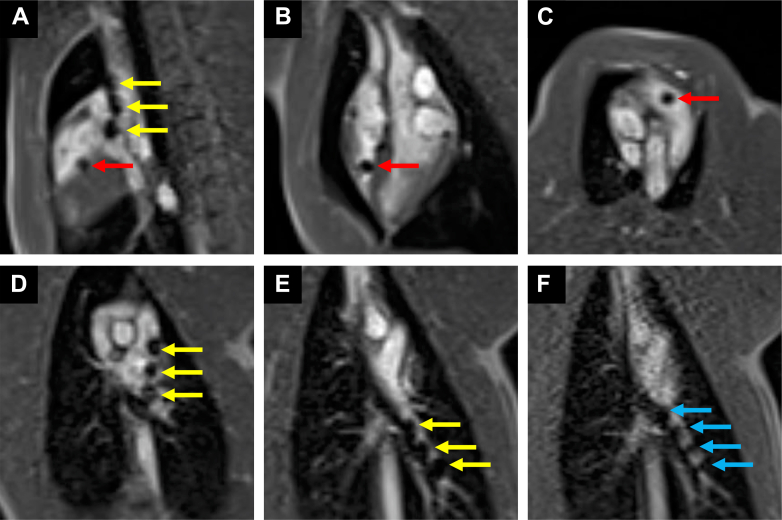
Guidewire and Wedge Catheter Navigation to the Left Pulmonary Artery Images were acquired with single-plane interactive real-time sequence. (A) The EmeryGlide guidewire and wedge catheter enter from the superior vena cava and bend into the right atrium. (B) The air-filled wedge catheter balloon reaches the apex of the right ventricle. (C) The air-filled wedge catheter balloon advances to the right ventricular outflow tract. (D) The guidewire passes through the main pulmonary artery and enters the left pulmonary artery. (E) The guidewire reaches the distal pulmonary artery. (F) The EmeryGlide guidewire is removed while the wedge catheter remains in place. A longer and stiffer Cook Medical prototype exchange length guidewire with 4 magnetic resonance–visible passive markers is then inserted. Magnetic resonance–visible markers on the EmeryGlide and Cook Medical guidewire are labeled with yellow and blue arrows, respectively. The air-filled balloon at the tip of the wedge catheter is labeled with a red arrow.

**Figure 3:**
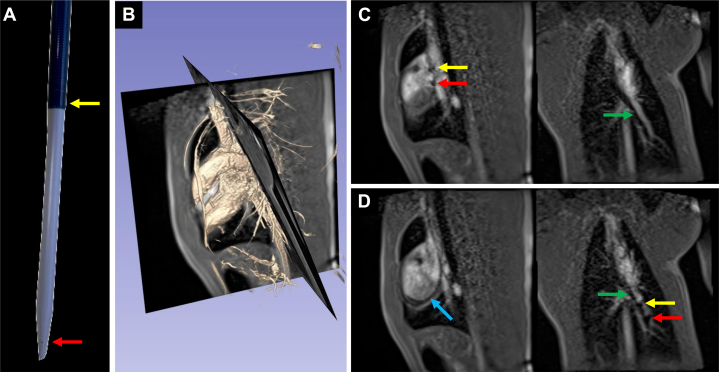
Sheath and Dilator with Magnetic Resonance–Visible Markers Advanced Over the Exchange Length Guidewire (A) An image of the sheath and dilator. (B) Corresponding imaging planes overlaid on a 3-dimensional rendering of the volumetric interpolated breath-hold examination Dixon scan. (C) The tip of the sheath and dilator positioned in the superior vena cava. (D) The tip of the sheath and dilator positioned in the left pulmonary artery. The markers at the tip of the sheath and dilator are labeled with yellow and red arrows, respectively. The shaft of the sheath is also visible and labeled with a blue arrow in panel D. Airways are labeled with green arrows. Images in panels C and D were acquired using a dual plane interactive real-time sequence.

Real-time MRI provided sufficient visualization to guide catheter navigation in all animals, regardless of access site. The advancement of the EmeryGlide guidewire and wedge catheter into the target branch PA was successfully performed under interactive real-time MRI guidance. The passive markers on the guidewire and the CO_2_-filled wedge catheter balloon were clearly visible in all MRI sequences (Figure 2, Video 1). When using external jugular venous access, once the guidewire and the wedge catheter reached the target, interleaved bicaval and branch PA imaging facilitated the exchange of the EmeryGlide guidewire for the MR-visible exchange length guidewire, followed by the advancement of the Flexor sheath and dilator. Passive markers on the exchange length guidewire (Figure 2, Video 2) and Flexor sheath and dilator were clearly visible (Figure 3, Video 3). After the sheath was positioned, the dilator was removed, leaving the sheath in place.

**Figure 4:**
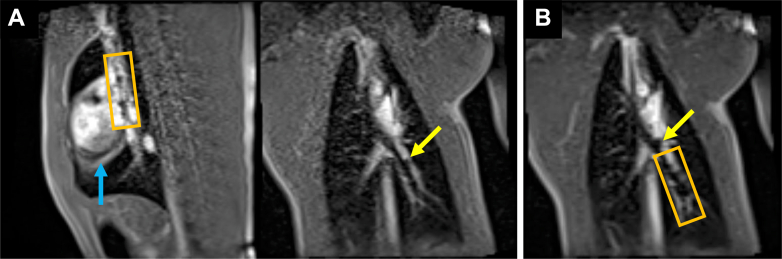
Stent and Balloon Advancement Through the Sheath (A) The stent and balloon are visualized in the superior vena cava view with dual-plane interactive real-time sequence, while the passive markers at the tip of the sheath are seen in the left pulmonary artery view during dual-plane acquisition. (B) After switching to a single-plane interactive real-time sequence, the stent has reached the left pulmonary artery and the sheath has been retracted to expose it. The stent is outlined with an orange box. The shaft of the sheath is labeled with a blue arrow and the passive marker at the sheath tip is labeled with a yellow arrow.

**Figure 5:**
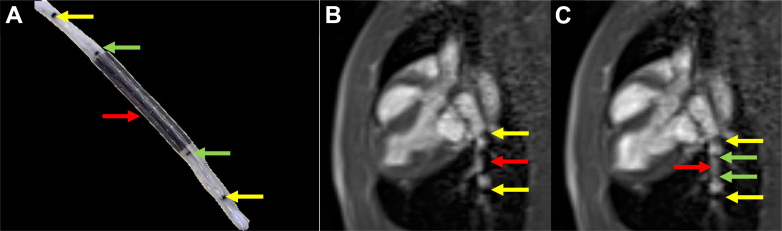
Stent Deployment in the Left Pulmonary Artery (A) An image of the stent crimped onto the balloon. The markers at the balloon tips are labeled with yellow arrows, the markers at the balloon shoulders with green arrows, and the stent with a red arrow. (B) A 26 mm Mega LD stent was crimped onto a 16 mm × 3 cm Z-Med custom balloon with 4 Nano4Imaging markers: 2 at the shoulders (0.5 mm, 1 layer thick) and 2 at the balloon tips (0.5 mm, 3 layers thick). An oblique sagittal plane shows the left pulmonary artery. (C) The balloon was inflated with a saline solution containing 1% gadolinium to deploy the stent, making the markers at the balloon shoulders visible. Images in panels B and C were acquired using a single-plane interactive real-time sequence.

Stainless steel stents crimped onto marked angioplasty balloons were advanced through the sheath to the target site (Figure 4, Video 4). The sheath was then retracted to expose the stent and balloon (Figure 4, Video 5). Two passive markers at the balloon tips were clearly visible, but the two passive markers at the balloon shoulders were obscured by the undeployed stent. All 4 markers on the balloon became visible during inflation (Figure 5, Video 6). The median total procedural time from wedge catheter insertion to stent deployment was 42 minutes, with a range of 30 minutes to 2 hours and 18 minutes, which was observed in the first successful case, reflecting the initial learning curve.

**Figure 6:**
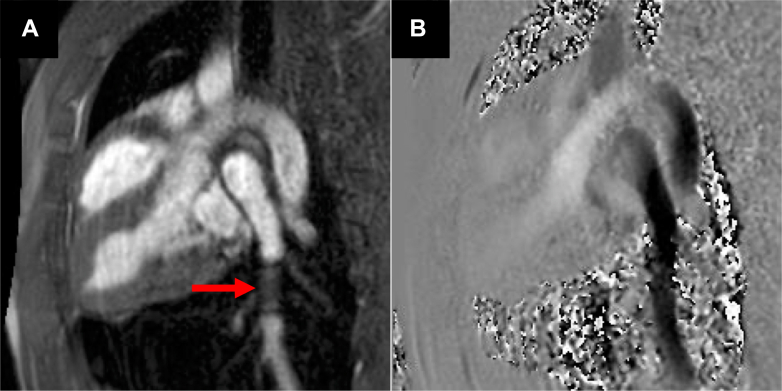
In-Plane Phase Contrast Spoiled Gradient Echo Images After Stent Deployment (A) Magnitude and (B) phase images acquired using in-plane phase contrast GRE at the level of the stented left pulmonary artery. A red arrow points to the stent in A. Flow continuity is preserved on the phase image B, and the vessel lumen remains sufficiently visualized to assess patency.

After balloon deflation and removal, postdeployment imaging confirmed successful stent placement. In-plane velocity mapping along the branch PA confirmed flow continuity (Figure 6, Video 7). Through-plane velocity mapping results were obtained in 4 pigs. In these animals, peak velocity distal to the stent (103.3 ± 24.5 cm/s) was on average 21.2 ± 7.0 cm/s higher than proximal to the stent (82.1 ± 18.1 cm/s), corresponding to a trivial pressure gradient of 0.2 ± 0.12 mm Hg, indicating no narrowing. The detailed analysis of proximal and distal velocity measurements, along with heart rate at the time of imaging to account for interanimal variation, is shown in Table 3.

**Table 3:** Peak Velocity Quantification From Through-Plane Phase Contrast MRI Performed Proximal and Distal to the Stent

	Peak Velocity (cm/s)		Heart Rate (beats/min)	
	Proximal	Distal	Proximal	Distal
Pig 5	84	96	124	116
Pig 8	59.7	76.3	117	119
Pig 9	104	135	82	80
Pig 10	80.8	106	131	132

Heart rate at the time of imaging is reported to account for interanimal variation.

MRI = magnetic resonance imaging.

## Discussion

iCMR offers significant advantages for catheter-based procedures in patients with CHD, notably eliminating harmful ionizing radiation for both patients and interventional teams and providing superior soft tissue contrast and quantitative cardiac function and blood flow assessment. During the decades of iCMR development at 1.5-T, the technology has been adopted clinically by a limited number of specialized centers worldwide,^2^^,^^9^^,^^15^, ^16^, ^17^, ^18^, ^19^, ^20^, ^21^, ^22^, ^23^, ^24^ as detailed in a recent state-of-the-art review.^6^ The challenging ergonomics posed by 1.5-T scanners with long, narrow bores and the lack of safe and easily visible devices have slowed the adoption of iCMR. The emergence of mid-field, wide-bore scanners has generated renewed interest, as the wider bore improves patient access and lower field strength reduces device heating and susceptibility artifacts, enabling the use of many commercially available catheterization devices with minor modifications. Recently, investigators at the National Institutes of Health reported the feasibility of diagnostic right heart catheterization in 7 patients using a higher-performance prototype 0.55-T magnetic resonance scanner, demonstrating the potential of iCMR at 0.55-T.^9^

This study extends our prior work and represents a pivotal step forward in iCMR by establishing the technical feasibility of real-time MRI-guided branch PA stenting at 0.55-T using commercially available devices with only minor modifications. Consistent with established workflows at 1.5-T, MR-visible passive markers on guidewires, sheaths, dilators, and balloons provided good visualization of equipment at 0.55-T, enabling the entire intervention to be performed without fluoroscopy. The reduced device heating at 0.55-T should allow clinicians to safely use many standard catheters, guidewires, and stents, streamlining the transition to MRI-guided procedures and broadening access to advanced interventions without the need for highly specialized equipment. The clinical implications of these findings are substantial, particularly for younger patients who face repeated exposure to ionizing radiation from conventional fluoroscopy interventions.

PA angioplasty and stenting are common interventions performed in patients with CHD and require navigation of stiff wires and long sheaths through complex cardiac anatomy to the branch PAs.^11^^,^^12^ Visualization is challenging with 2-dimensional MRI, as devices frequently move in and out of the imaging plane. The adaptation from femoral to external jugular venous access, which resulted in a 100% success rate for stent deployment in subsequent cases, highlights the importance of procedural flexibility and iterative workflow optimization in clinical translation.

The modified devices used in these procedures require additional improvements before they can be deployed for clinical iCMR procedures. For example, the MR-visible exchange length guidewire is a hydrophilic wire. While the stiffness was adequate for branch PA stenting with large-diameter stents that can be dilated to adult size (≥18 mm), the hydrophilic coating made sheath and balloon catheter exchanges difficult, due to poor tactility. The entire length of the wire shaft also was not visible, particularly when aligned parallel to the imaging field. However, the sheath and dilator were clearly visible along their entire lengths. The commercially available Max and Mega LD stainless steel stents also had good visibility at 0.55-T without exaggerated artifact, and they did not require any modifications. The passive markers on the tips of the balloon could be distinguished well from the stent itself, but the markers on the shoulders, which is the typical location for balloon markers, were obscured by the metal susceptibility artifact surrounding the stent. The markers on the tips are necessary to verify that the crimped stent has not slipped on the balloon prior to inflation. This procedure requires multiple devices with passive markers, all of which appear as black artifacts. Multiple overlapping markers can create confusion as to the location of the different portions of devices; this is a challenge that must be overcome prior to bringing this technology to patients. Using artificial intelligence to identify and color-code markers is one potential solution.^25^^,^^26^

The commercially available 0.55-T scanner used in this study has reduced gradient performance and lower intrinsic SNR compared with higher-field systems, limiting temporal and spatial resolution. Sequence parameters were optimized to balance these constraints, achieving frame rates sufficient for guidance (4.3 fps for single-plane and 2.6 fps for dual-plane acquisition). Unlike fluoroscopy, which provides continuous projection imaging, the tomographic slices acquired by MRI can only visualize devices within the imaging plane. Dual-plane imaging strategies, though reducing frame rate, allowed simultaneous monitoring of sheath advancement and wire navigation. Guttman et al^27^ previously described multislice interleaved acquisitions for MRI-guided cardiovascular interventions, including endograft repair of aortic aneurysm, balloon septostomy of the cardiac interatrial septum, angioplasty and stenting, and endomyocardial cell injection. Notably, in that study, the interleaved imaging captured guidewire prolapse into the IVC during sheath manipulation, enabling prompt identification and corrective action. While imaging in multiple planes simultaneously decreased the frame rate, in our experience, it still proved fast enough to guide the balloon catheter/stent complex and sheath.

Procedural times varied widely, reflecting both the learning curve and technical challenges of identifying all the various passive markers on multiple pieces of catheterization equipment used together. In the early successful cases, additional time was needed to explore and refine procedural workflows and optimize imaging strategies. For future training purposes, we recommend starting with static phantom sessions to allow the interventional cardiologist, radiologist, and MRI technician to become familiar with the setup and interactive scanning controls before progressing to in vivo experiments.

Mid-field MRI offers reduced susceptibility artifacts from metal implants,^28^^,^^29^ allowing clear visualization of the vessel lumen and wall poststenting and immediate confirmation of stent placement and vessel patency. Quantitative flow measurements and calculated pressure gradients were assessed intraprocedurally, supporting rapid clinical decision making and potentially improving patient outcomes. However, further validation of these hemodynamic metrics is needed.

While we successfully demonstrated the feasibility of performing a complex interventional procedure with mid-field MRI guidance, ergonomic challenges remain, as leaning into the bore may offset the advantage of not wearing lead aprons. Redesigning the bore and table for interventional procedures is critical. Device safety at 0.55-T must be carefully evaluated before clinical testing, as reduced heating cannot be assumed for all device geometries.^30^ In this feasibility study, we did not perform real-time temperature monitoring. While the subjects in the successful cohort survived until the end of the experiment without adverse clinical events, formal safety testing in phantoms (ASTM F2182) is required to quantify heating risks prior to human use.^31^ The modified devices and imaging techniques also require further refinement to identify overlapping and multiple passive markers, especially when multiple devices are deployed simultaneously. The interventional wire needs to be hydrophobic, and the shaft should be visible throughout its length. Temporal and spatial resolution constraints occasionally limited real-time imaging performance, especially during rapid device manipulation. To maintain acceptable temporal resolution, we used relatively coarse spatial resolution, which introduced partial volume effects and blurred vessel boundaries. Advanced acquisition techniques, such as radial or spiral k-space trajectories and deep learning–based reconstruction and denoising,^32^, ^33^, ^34^ may improve frame rates and image quality. Continued refinement of device design, marker visibility, and imaging sequences will be essential to match the usability of fluoroscopy and ensure reproducibility. One animal expired prior to the procedure. While the animal did not undergo postmortem examination, the clinical presentation was consistent with a hypersensitivity reaction to ferumoxytol (a known issue in the Yorkshire swine breed), rather than a procedural complication related to the MRI guidance.^35^ Finally, we did not compare imaging performance or workflow to higher field systems; however, the primary goal of this study was to establish feasibility at mid field rather than to assess comparative performance.

## Conclusions

This study shows the technical feasibility of stenting branch PAs with large-diameter stainless steel balloon-expandable stents using mid-field MRI guidance and commercially available devices with some minor modifications. Mid-field scanners may be the catalyst needed for broader clinical acceptance of iCMR; however, continued collaboration among industry, academic researchers, and clinicians will be essential for successful translation into clinical routine.

Perspectives **COMPETENCY IN MEDICAL KNOWLEDGE:** This preclinical study demonstrates that real-time MRI-guided branch PA stenting can be performed at 0.55-T using commercially available equipment with only minor modifications. Clinicians should recognize the high radiation burden associated with conventional PA angioplasty and stenting in congenital heart disease, in which patients often require multiple lifetime catheterizations. Understanding that mid-field MRI reduces radiofrequency-induced device heating is critical because it may enable the safe use of standard catheters, guidewires, and stainless steel stents during interventional procedures. This work shows that real-time MRI can reliably visualize devices, confirm stent position, and assess flow immediately after deployment, supporting MRI as a viable alternative to fluoroscopy for selected vascular interventions. These findings broaden the procedural toolkit available to interventionalists and highlight the growing role of mid-field MRI in radiation-free cardiovascular interventions.

**TRANSLATIONAL OUTLOOK:** Before MRI-guided PA stenting can be applied clinically, several technical and workflow challenges must be addressed. Improved device design is needed to streamline procedural navigation, including hydrophobic wires, enhanced shaft visibility, and passive marker patterns that remain interpretable when overlapping. Advances in real-time MRI sequences, multiplane visualization, and faster acquisition strategies will be essential to match the usability of fluoroscopy. Rigorous device-heating assessments, standardized protocols, and operator training pathways are also required to ensure safety and reproducibility. Future human studies will determine how mid-field iCMR can be integrated into routine congenital catheterization practice and whether it can meaningfully reduce cumulative radiation exposure in this high-risk population.

## Funding Support and Author Disclosures

Research reported in this publication was supported by the National Heart, Lung, and Blood Institute of the National Institutes of Health under award number R01HL161618. Drs Jin and Pang are employees of Siemens Medical Solutions USA, Inc. Drs Maier and Krafft are employees of Siemens Healthineers GmbH. Mr Ooms and Dr Roll are employees of Cook Advanced Technologies. Mr Kreiger is an employee of Cook Medical. Dr Gross and Mr Bramwell are employees of MED Institute. Mr de Mos is an employee of Nano4Imaging GmbH. Dr Borm is a shareholder of Nano4Imaging. Y. Liu was supported by the American Heart Association and Children’s Heart Foundation. Dr Simonetti has received institutional research support from Siemens Healthineers. Dr Armstrong has served as a proctor Medtronic, Edwards Lifesciences, Abbott, B. Braun Interventional Systems, and Renata Medical; as a consultant for Medtronic, Edwards Lifesciences, Abbott, B. Braun Interventional Systems, Renata Medical, Starlight Cardiovascular, and Cook Medical and as an investigator for Edwards Lifesciences, Renata Medical, Starlight Cardiovascular, Autus Valve Technologies, atHeart Medical, and Merit Medical. All other authors have reported that they have no relationships relevant to the contents of this paper to disclose.

## Appendix

Video 1 Guidewire and wedge catheter navigation to the left pulmonary artery. Images were acquired with single plane IRT sequence. A yellow arrow tracks the EmeryGlide guidewire, and a red arrow tracks the wedge catheter. The EmeryGlide guidewire and wedge catheter enter from the superior vena cava and bend into the right atrium. The air-filled wedge catheter balloon reaches the apex of the right ventricle. The air-filled wedge catheter balloon advances to the RVOT. The guidewire passes through the MPA and enters the LPA. The guidewire reaches the distal pulmonary artery. This video links to Figure 2.

Video 2 Guidewire and wedge catheter navigation to the left pulmonary artery. The EmeryGlide guidewire is removed while the wedge catheter remains in place. A longer and stiffer Cook Medical prototype exchange length guidewire with 4 MR-visible passive markers, is then inserted. A blue arrow tracks the exchange length guidewire. This video links to Figure 2F.

Video 3 Sheath and dilator with MR-visible markers advanced over the exchange length guidewire. Images were acquired using a dual plane IRT sequence. The tip of the sheath and dilator positioned in the SVC. The tip of the sheath and dilator positioned in the LPA. The shaft of the sheath is also visible. A red arrow tracks the sheath and dilator. This video links to Figure 3.

Video 4 Stent and balloon advancement through the sheath. Images were acquired using a dual plane IRT sequence. The stent and balloon are visualized in the SVC view, while the passive markers at the tip of the sheath are seen in the LPA view. A yellow arrow tracks the stent and balloon. This video links to Figure 4A.

Video 5 Retract the sheath to expose stent and balloon. Images were acquired using a single plane IRT sequence. The stent has reached the LPA. The sheath has been retracted to expose the stent and balloon. A red arrow tracks the sheath tip as it is retracted. This video links to Figure 4B.

Video 6 Stent deployment in the left pulmonary artery. Images were acquired using a single plane IRT sequence. An oblique sagittal plane shows the LPA. The balloon was inflated with a saline solution containing 1% gadolinium to deploy the stent, making the markers at the balloon shoulders visible. A red arrow labels the stent. This video links to Figure 5.

Video 7 Combined magnitude and phase in-plane phase-contrast GRE images after stent deployment. Left: Magnitude images showing the stent in the left pulmonary artery. Right: Phase images showing preserved flow continuity through the stent. This video links to Figure 6.
